# Diagnostic performance of interleukin-27 and C-reactive protein in neonatal sepsis: an updated systematic review and meta-analysis

**DOI:** 10.3389/fped.2026.1781391

**Published:** 2026-05-13

**Authors:** Wei Liu, Yan Zhang, Weidong Liu, Jun Luo

**Affiliations:** Department of Neonatology, Shenzhen Baoan Women's and Children's Hospital, Guangdong, China

**Keywords:** crp, interleukin-27, meta-analysis, neonatal sepsis, neonate

## Abstract

**Background:**

Interleukin-27 (IL-27) has been proposed as a promising diagnosis marker for neonatal sepsis. However, findings across existing clinical studies remain inconsistent and inconclusive. This study aims to comprehensively evaluate and compare the diagnostic accuracy of IL-27 vs. C-reactive protein (CRP) for neonatal sepsis, based on the latest available evidence.

**Methods:**

This systematic review and meta-analysis was conducted following the PRISMA guidelines and prospectively registered in PROSPERO (CRD420251272080). PubMed, Embase, and the Cochrane Library were searched for relevant studies published from January 1996 to December 2025. Eligible studies were collected and analyzed using Review Manager 5.4 and R software. Meta-Disc 1.4 was applied to calculate pooled sensitivity, specificity, and summary receiver operating characteristic (SROC) curves with corresponding area under the curve (AUC) values to evaluate overall diagnostic efficacy.

**Results:**

A total of five controlled studies involving 495 neonates were ultimately included in the final analysis, all of which were judged to have acceptable methodological quality. Meta-analysis results showed that the pooled sensitivity of IL-27 for diagnosing neonatal sepsis was 0.82 [95% confidence interval (CI), 0.77–0.87], which was significantly higher than that of CRP (0.73; 95% CI, 0.65–0.79). The pooled specificity of IL-27 was 0.85 (95% CI, 0.80–0.90), also exceeding that of CRP (0.76; 95% CI, 0.69–0.83). SROC curve analysis further demonstrated robust predictive value for both biomarkers, with the AUC of IL-27 (0.92) being notably higher than that of CRP (0.84).

**Conclusions:**

The findings of this updated systematic review and meta-analysis indicate that IL-27 has superior diagnostic accuracy to CRP for the identification of neonatal sepsis. IL-27 may therefore serve as a more reliable biomarker to assist in the early and accurate diagnosis of neonatal sepsis in clinical practice.

**Systematic Review Registration:**

https://www.crd.york.ac.uk/PROSPERO/, identifier CRD420251272080.

## 1 Introduction

Despite recent advances in neonatal intensive care, sepsis remains a leading cause of morbidity and mortality, especially among preterm infants (1, 2). The prevalence of sepsis-associated morbidity and mortality in neonates varies widely, ranging from 5.6% to 27.3% and 12.5% to 24.0%, respectively (3, 4). Early diagnosis and targeted intervention are critical to improving clinical outcomes; however, this goal remains challenging due to the nonspecific clinical manifestations of neonatal sepsis. Clinicians thus continue to seek reliable biomarkers that can facilitate timely and accurate identification of the condition, thereby guiding appropriate antibiotic therapy and reducing the risk of adverse events.

Interleukin-27 (IL-27), a member of the IL-6/IL-12 cytokine family first characterized in 2001, exerts diverse modulatory effects on immune responses during infectious processes (5). Emerging evidence indicates that IL-27 impairs macrophage phagocytic function in sepsis by exacerbating mitochondrial dysfunction (6, 7), and circulating levels of this pro-inflammatory cytokine are elevated in critically ill patients with sepsis admitted to the intensive care unit (ICU) (8). A seminal study by Tosson et al. (9) demonstrated that IL-27 serves as a highly effective biomarker for neonatal sepsis, with diagnostic performance superior to that of mean platelet volume (MPV): at a cutoff value of 283.8 pg/mL, IL-27 achieved a sensitivity of 97.8% and a specificity of 100%. However, conflicting findings have emerged from subsequent research, with some studies reporting that the diagnostic utility of IL-27 is inferior to that of other inflammatory markers, including C-reactive protein (CRP) and procalcitonin (10, 11). Given these discrepant results, a comprehensive updated review of the latest evidence is urgently needed to specifically compare the diagnostic accuracy of IL-27 and CRP in neonatal sepsis—an important gap that has not been fully addressed in prior analyses focusing on broader pediatric populations.

Accordingly, we conducted a systematic review and meta-analysis to synthesize all available evidence on the diagnostic performance of IL-27 and CRP for neonatal sepsis. Our primary objective was to systematically and quantitatively evaluate published studies, thereby clarifying the relative utility of these two biomarkers for the early identification of sepsis in this vulnerable population.

## 2 Methods

### 2.1 Literature search and study selection

A comprehensive computerized literature search was performed across three electronic databases: PUBMED, EMBASE (http://www.embase.com/) and the Cochrane Library (http://www.the-cochranelibrary.com/view/0/index.html). The search covered publications from January 1996 to December 2025, with no restrictions on study design except those specified in the inclusion criteria. This systematic review and meta-analysis was prospectively registered in the International Prospective Register of Systematic Reviews (PROSPERO; registration ID: CRD420251272080; available at https://www.crd.york.ac.uk/PROSPERO/).

The search strategy combined subject terms and free-text keywords related to the target biomarker and disease, including: “*Interleukin-27*,” “*IL-27*,” “*sepsis*,” “*neonate*,” “*newborn*,” “*infant*,” “*preterm*,” “*premature*,” “*Neonatal Sepsis,” “Neonatal Early-Onset Sepsis,” “Neonatal Early Onset Sepsis,” “Neonatal Late Onset Sepsis,” “Neonatal Late-Onset Sepsis,”* along with their mutual combinations. To ensure comprehensive literature coverage, the reference lists of all included studies and relevant narrative reviews were manually screened for additional eligible publications.

Studies were included in the meta-analysis if they met all the following criteria: (1) Study design: observational or interventional studies focusing on diagnostic test accuracy; (2) Exposure measure: assessment of circulating IL-27 and/or CRP levels in neonates; (3) Study population: neonates with confirmed sepsis assigned to the experimental group, and neonates suspected of sepsis but with a definitive non-sepsis diagnosis assigned to the control group; (4) Data availability: sufficient raw data to calculate diagnostic metrics, including true positives (TP), false positives (FP), true negatives (TN), and false negatives (FN); (5) Timing of measurement: IL-27 blood sampling performed at the time of clinical presentation with suspected sepsis, prior to the initiation of antimicrobial therapy, or in asymptomatic neonates at study enrollment; and (6) Outcome definition: sepsis confirmed by positive microbial blood culture, consistent across all included studies. Neonatal sepsis was defined as a positive microbial blood culture in the studies reviewed. Studies were excluded if they met any of the following criteria: (1) Study type: abstracts, reviews, case reports, commentaries, or animal experimental studies; (2) Biomarker scope: diagnostic evaluations that did not include IL-27; (3) Data inadequacy: insufficient information to extract or calculate TP, FP, TN, and FN values; (4) Study nature: bioinformatics analyses, in silico studies, or duplicate publications (only the most recent or comprehensive dataset was retained); and (5) Language restriction: studies not published in English. Two independent investigators conducted the literature screening, title and abstract review, and full-text eligibility assessment. Any discrepancies were resolved through consensus; if no agreement was reached, a third senior investigator was consulted to make the final decision.

### 2.2 Data extraction

A standardized data extraction form was developed and piloted before formal use. Two reviewers independently extracted the following key information from each eligible study: (1) Basic study characteristics: title, publication year, study design, and geographic region; (2) Study population details: sepsis onset type (early-onset vs. late-onset), gestational age, postnatal age, sample size of experimental and control groups; (3) Biomarker assay parameters: method of IL-27 and CRP measurement, cutoff values used for diagnostic thresholding, and timing of blood sample collection; (4) Diagnostic outcome data: TP, FP, TN, and FN counts for both IL-27 and CRP. Discrepancies between the two reviewers were resolved via discussion; if consensus could not be achieved, a third reviewer adjudicated the final data extraction results.

### 2.3 Methodological quality assessment

The methodological quality of included diagnostic accuracy studies was independently evaluated by two reviewers using the Quality Assessment of Diagnostic Accuracy Studies 2 (QUADAS-2) tool, in accordance with the guidelines outlined in the Cochrane Handbook for Diagnostic Test Accuracy Reviews. Each domain of QUADAS-2 (patient selection, index test, reference standard, flow and timing) was rated as low risk of bias, high risk of bias, or unclear risk of bias (12). Any disagreements in quality ratings were resolved through consensus or consultation with a third reviewer.

### 2.4 Statistical analysis

All statistical analyses were performed using Review Manager (RevMan) 5.4, Meta-DiSc 1.4, and R software. The summary receiver operating characteristic (SROC) curve was constructed to evaluate the overall diagnostic performance of IL-27 and CRP for neonatal sepsis, with the area under the SROC curve (AUC) calculated to quantify discriminative ability.

Heterogeneity across included studies was assessed using the Cochran *Q*-test and I^2^ statistic. The *I*^2^ statistic ranges from 0% to 100%, with values ≥50% combined with a Cochran *Q*-test *P*-value < 0.05 indicating substantial heterogeneity (13). In the presence of significant heterogeneity, potential sources (e.g., study design, population characteristics, assay methods, cutoff values) were explored via subgroup analysis or sensitivity analysis where feasible. Publication bias was evaluated using Egger's test and Begg's test (14). A *P*-value < 0.05 was considered indicative of statistically significant publication bias.

## 3 Results

### 3.1 Characteristics of included studies

**Figure 1:**
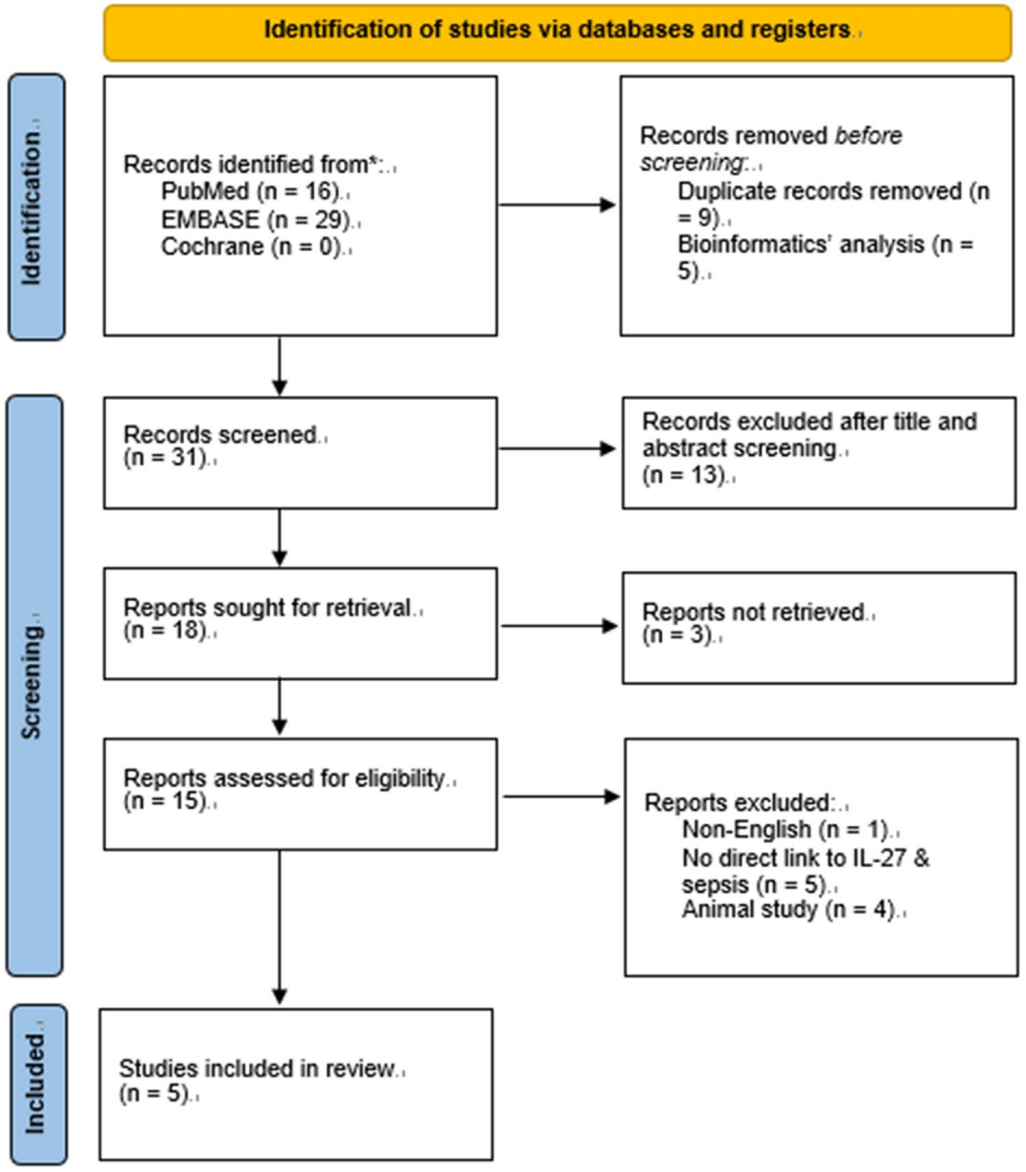
Flow chart of article screening and the selection process.

A total of 45 unique records were initially retrieved from the three electronic databases. After the removal of 14 duplicates and bioinformatics-focused studies, the remaining 31 records underwent title and abstract screening, with 13 irrelevant articles excluded at this stage. Subsequent full-text review eliminated studies that were non-English, animal-based, or lacked direct relevance to IL-27 and neonatal sepsis. Ultimately, five studies met all predefined inclusion criteria and were included in the final meta-analysis (9–11, 15, 16). The detailed literature selection process is depicted in Figure 1. Key characteristics and extracted data of the included studies are summarized in Table 1.

**Table 1:** Characteristics of 5 studies enrolled in the meta-analysis.

Reference	Region	Study design	Outcome	Sample size (Cases/Controls)	Age	Biomarker	Cut-off^a^	Measurement	Sensitivity (%)	Specificity (%)
Rautela (10)	India	Prospective	Culture-proven EOS	80 (41/39)	Neonate	IL-27	–	ELISA	78	62
						CRP	6	immunoturbidometry	73	79
Tosson (9)	Egypt	Prospective	Culture-proven LOS	90 (45/45)	Neonate (full-term)	IL-27	283.8	ELISA	98	100
Fahmy (15)	Egypt	Prospective	Culture-proven EOS	84 (47/37)	Neonate	IL-27	-	ELISA	81	84
Abo (16)	Egypt	Prospective	Culture-proven sepsis	90 (45/45)	Neonate	IL-27	485.56	ELISA	96	100
						CRP	32	immunoturbidometry	89	82
He (11)	China	Prospective	Culture-proven EOS	151 (68/83)	Neonate （late preterm and term）	IL-27	1,000	ELISA	71	71
						CRP	3	immunoturbidometry	68	66

EOS, early-onset sepsis; LOS, late-onset sepsis.

a IL-27, pg/mL; CRP, mg/dL.

### 3.2 Methodological quality assessment

**Figure 2:**
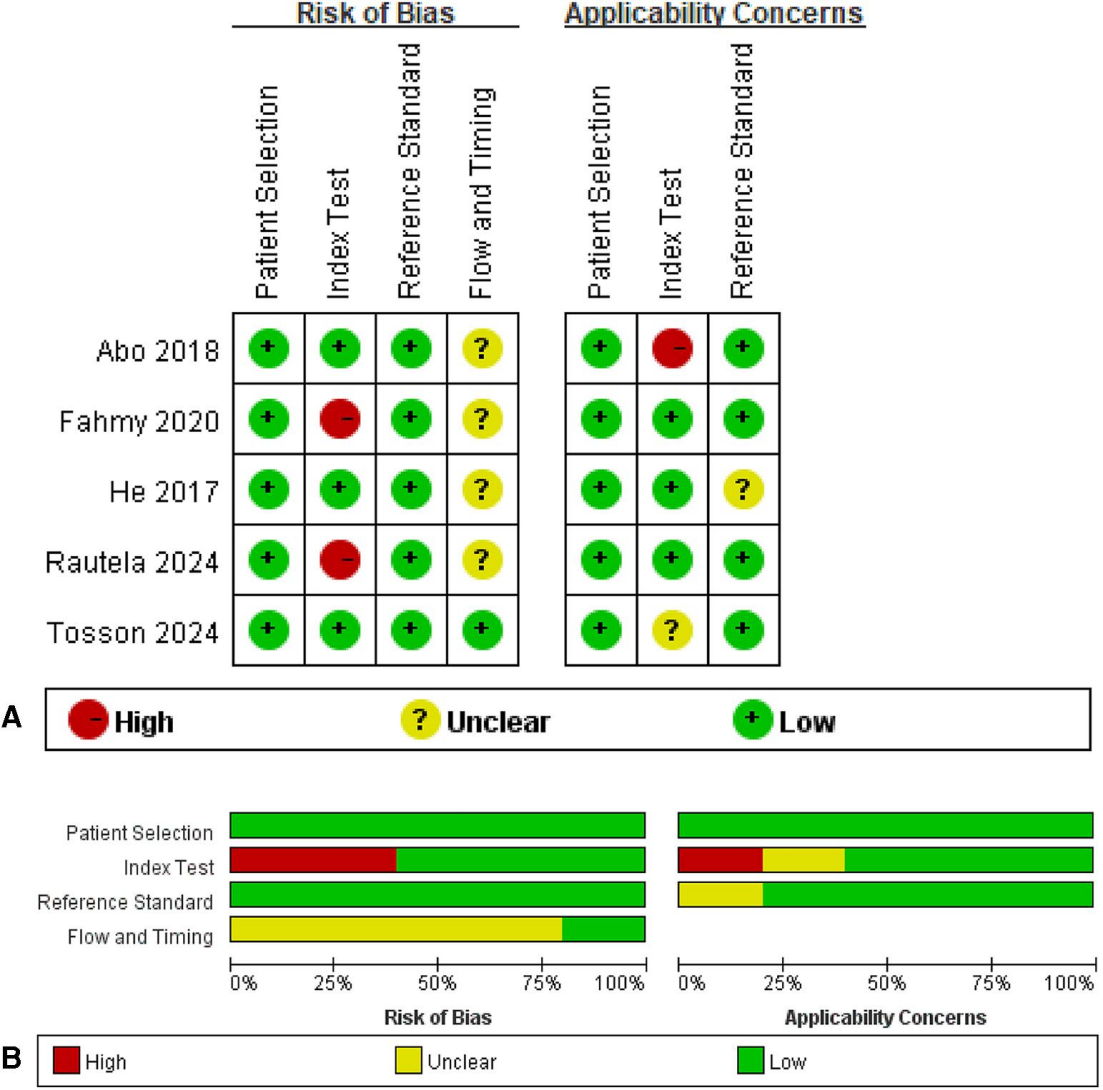
Risk of bias and applicability concerns summary: review authors' judgements about each domain for each included study. **(A)** Risk of bias in the included studies. **(B)** Evaluation of the risk of bias in the included studies on patients with sepsis.

Methodological quality assessment of the included studies was performed using the QUADAS-2 tool, and the results are presented in Figure 2. In this visualization, red circles indicate high risk of bias, green circles denote low risk of bias, and yellow circles represent unclear risk of bias. Unclear risk ratings were assigned when study reports lacked sufficient detail to permit definitive judgments on specific quality domains.

Specifically, in the index test domain, two studies were classified as having a high risk of bias due to the absence of a predefined diagnostic threshold for IL-27 (10, 15). For applicability concerns in this domain, one study was rated as high risk (16), while another was categorized as unclear risk (9). Regarding the flow and timing domain, four studies were deemed to have an unclear risk of bias (10, 11, 15, 16). For the reference standard domain, He et al. (11) was assessed as having an unclear risk of applicability concern.

### 3.3 Diagnostic accuracy of IL-27 and CRP

Meta-analysis was conducted to compare the diagnostic performance of IL-27 and CRP for neonatal sepsis across the five included studies. The results demonstrated that IL-27 had superior sensitivity and specificity relative to CRP for the diagnosis of neonatal sepsis.

**Figure 3:**
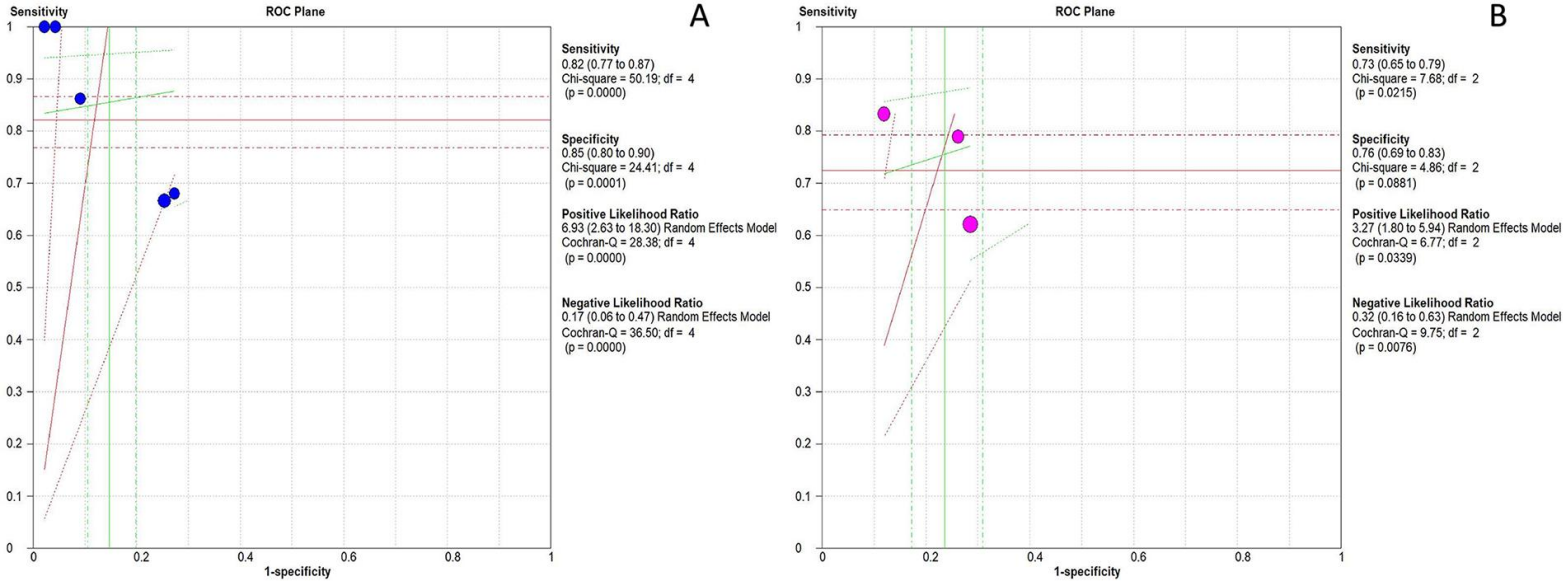
SROC curve for assessment of the diagnostic accuracy of IL-27 and CRP to predict sepsis in infants and children. **(A)** ROC plane for IL-27. **(B)** ROC plane for CRP.

**Figure 4:**
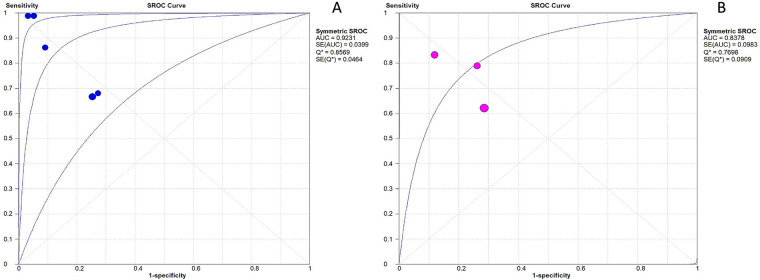
ROC plane for assessment of the diagnostic accuracy of IL-27 and CRP to predict sepsis in infants and children. **(A)** ROC plane for IL-27. **(B)** ROC plane for CRP.

Summary receiver operating characteristic (SROC) curves were constructed for both biomarkers. For IL-27, the area under the SROC curve (AUC) was 0.92 [95% confidence interval (CI): 0.87–0.97] (Figure 3). The pooled sensitivity of IL-27 was 0.82 (95% CI: 0.77–0.87), and the pooled specificity was 0.85 (95% CI: 0.80–0.90) (Figure 4). The positive likelihood ratio (LR+) of IL-27 was 6.93 (95% CI: 2.63–18.30), a magnitude sufficient to support its utility as a rule-in test for neonatal sepsis. In contrast, the negative likelihood ratio (LR-) was 0.17 (95% CI: 0.06–0.47), which was not low enough to safely exclude sepsis by reducing pretest probability to a clinically acceptable threshold.

For CRP, the AUC was 0.84 (95% CI: 0.80–0.88). The pooled sensitivity and specificity were 0.73 (95% CI: 0.65–0.79) and 0.76 (95% CI: 0.69–0.83), respectively (Figure 4). The LR+ of CRP was 3.27 (95% CI: 1.80–5.94), which could also support rule-in utility, while the LR- was 0.32 (95% CI: 0.16–0.63)—a value that similarly failed to enable safe exclusion of sepsis.

The diagnostic odds ratio (DOR) for IL-27 was 54.97 (95% CI: 8.71–347.13), whereas the DOR for CRP was 10.85 (95% CI: 3.10–37.94) (Figure 4), further confirming the superior diagnostic efficacy of IL-27.

### 3.4 Heterogeneity assessment

Forest plot analyses revealed substantial heterogeneity across the included studies for all key diagnostic metrics: sensitivity (*I*^2^ = 92.0%), specificity (*I*^2^ = 83.6%), positive LR (*I*^2^ = 85.9%), negative LR (*I*^2^ = 89.0%), and DOR (*I*^2^ = 88.4%). Visual inspection of study characteristics identified two potential sources of heterogeneity: variability in sepsis onset type (early-onset vs. late-onset) and a broad range of IL-27 cutoff values (283.8–1,000.0 pg/mL).

To explore these sources, subgroup analyses were performed based on sepsis onset timing [early-onset sepsis [EOS] vs. late-onset sepsis [LOS]] and geographic region (Egypt vs. non-Egypt); results are summarized in Table 2. Although these analyses should be interpreted with caution due to the small number of studies included in each subgroup, we observed the following trends.

**Table 2:** Subgroup analysis for assessment of the diagnostic accuracy of IL-27 to predict sepsis in infants.

Subgroup	No. of studies	Pooled sensitivity (%) (95% CI)	Pooled specificity (%) (95% CI)	Pooled LR+ (95% CI)^a^	Pooled LR− (95% CI)^b^	Pooled DOR 95% CI	*I*^2^ specificity %
Region (Egypt)	3	95 (90–98)	95 (90–98)	15.51 (7.56–31.80)	0.03 (0.00–0.55)	474.41 (31.74–7,089.93)	0
Region (Non-Egypt)	2	67 (58–76)	74 (65–82)	2.59 (7.56–31.80)	0.44 (0.34–0.59)	5.83 (3.29–10.33)	0
Timing of sepsis (EOS)	3	73 (66–79)	78 (70–84)	3.36 (1.79–6.31)	0.33 (0.19–0.59)	11.20 (3.23–38.90)	59
Timing of sepsis (LOS)	2	100 (96–100)	97 (91–99)	22.76 (8.73–59.32)	0.01 (0.00–0.08)	2,039.25 (220–18,819)	0

a LR+, positive likelihood ratio.

b LR−, negative likelihood ratio; EOS, early onset sepsis.

LOS, late onset sepsis.

By geographic region: Studies conducted in Egypt showed a numerically higher pooled sensitivity for IL-27 (95%, 95% CI: 90–98) than studies from other regions (67%, 95% CI: 58–76). Pooled specificity was also higher in Egyptian studies (95%, 95% CI: 90–98) than in non-Egyptian studies (74%, 95% CI: 65–82), with corresponding DOR values of 474.41 (95% CI: 31.74–7,090) and 5.83 (95% CI: 3.29–10.33), respectively.

By sepsis onset timing: Studies focusing on LOS exhibited a higher pooled sensitivity (100%, 95% CI: 96–100) than those focusing on EOS (73%, 95% CI: 66–79). Pooled specificity was also significantly higher in LOS studies (97%, 95% CI: 91–99) compared with EOS studies (78%, 95% CI: 70–84), with corresponding *I*^2^ values for specificity of 59% and 0%, respectively.

### 3.5 Publication bias

**Figure 5:**
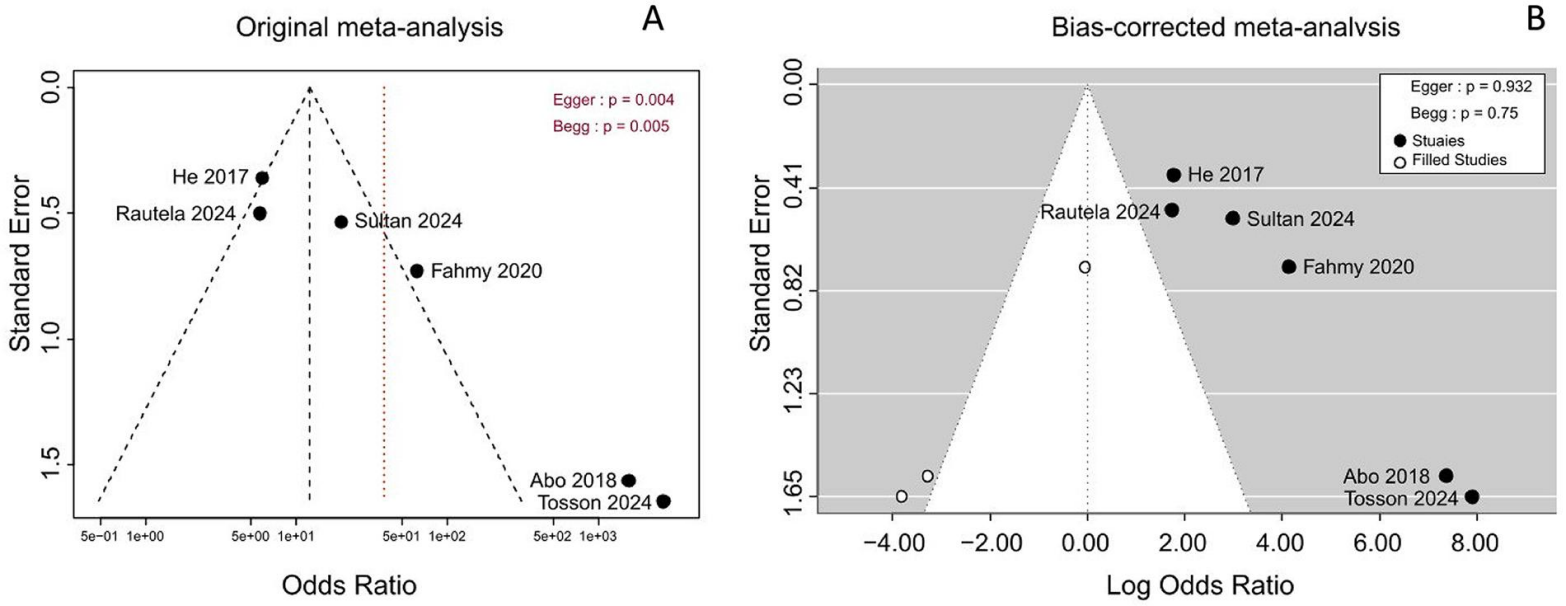
The publication bias was evaluated by Egger's test and Begg's test. **(A)** Original analysis. **(B)** Bias-corrected analysis.

Potential publication bias among the included studies was evaluated using Egger's test and Begg's test, with funnel plot asymmetry (Figure 5A) used to complement the quantitative assessment. The results showed statistically significant publication bias, with *P*-values of 0.004 (Egger's test) and 0.005 (Begg's test), respectively. However, bias-corrected analyses mitigated this concern: the corrected *P*-values were 0.93 (Egger's test) and 0.75 (Begg's test), indicating no evidence of publication bias after correction (Figure 5B).

## 4 Discussion

Early diagnosis and timely initiation of targeted therapy are pivotal for improving outcomes in neonatal sepsis, a leading cause of morbidity and mortality in neonates despite advances in intensive care. Identifying rapid, sensitive, and specific biomarkers is therefore critical to optimizing clinical management. The findings of this meta-analysis demonstrate that IL-27 has superior diagnostic accuracy for neonatal sepsis compared with CRP, a well-established inflammatory marker. Specifically, IL-27 exhibited a pooled sensitivity of 0.82 (95% CI, 0.77–0.87) and specificity of 0.85 (95% CI, 0.80–0.90), with an area under the SROC curve (AUC) of 0.92—indicative of robust predictive performance. To our knowledge, this is the first systematic review and meta-analysis to specifically evaluate the diagnostic utility of IL-27 in neonatal sepsis and directly compare its performance with CRP, addressing a critical gap in the existing literature.

IL-27, a heterodimeric cytokine composed of IL-27-p28 and EBI3 subunits, belongs to the IL-6/IL-12 family and plays a key modulatory role in inflammatory and immune responses. Produced by antigen-presenting cells upon stimulation by microbial products or inflammatory cues, IL-27 regulates T-cell function with both pro- and anti-inflammatory properties (5, 17). Preclinical studies have highlighted its relevance to sepsis pathophysiology: IL-27 is rapidly induced in murine models of septic peritonitis, and genetic ablation of EBI3 or neutralization of IL-27 confers protective effects in these models (18). A recent study further identified a direct link between IL-27 signaling and bacterial killing, suggesting potential therapeutic value of IL-27 antagonism as a host-directed therapy for neonates (19). Mechanistically, IL-27 exerts its effects in macrophages primarily through signal transducer and activator of transcription (STAT)-1 or STAT-3; however, STAT-3 is dispensable for IL-27-mediated macrophage suppression and does not represent a viable therapeutic target in Escherichia coli-induced neonatal sepsis (20). Despite these insights, the precise clinical implications of IL-27 in neonatal sepsis remain incompletely understood, underscoring the need for further translational research.

Several studies have reported IL-27 as a promising biomarker for early identification of neonatal sepsis (21, 22). Tosson et al. demonstrated that an IL-27 cutoff value of 283.8 pg/mL yielded exceptional sensitivity (97.8%) and specificity (100%) for diagnosing late-onset neonatal sepsis (LOS) (9). Fahmy et al. similarly showed that IL-27 levels effectively identified early-onset neonatal sepsis (EOS) on the first day of life, with a sensitivity of 0.936 and specificity of 0.811 (15); notably, IL-27 was an independent predictor of EOS in multivariate analysis, and its combination with other inflammatory markers (e.g., IL-6, Tumor Necrosis Factor-alpha [TNF-α], Heat Shock Protein 70 [HSP70], Matrix Metalloproteinase-8 [MMP-8], procalcitonin [PCT], and CRP) further enhanced predictive accuracy (11). In contrast, Rautela et al. reported more modest diagnostic performance for IL-27 (sensitivity: 78.05%, specificity: 61.54%) but noted comparable sensitivity and negative predictive value (NPV) to PCT, albeit with lower specificity than CRP (10). Collectively, these studies—along with our meta-analytic results—support IL-27 as a reliable biomarker for neonatal sepsis, with performance that is at least comparable to, and often superior to, conventional markers.

Diagnostic accuracy tests typically involve a trade-off between sensitivity and specificity, where increased true positive rates may be accompanied by decreased true negative rates. Thus, relying solely on these two metrics may not fully capture overall diagnostic performance. The AUC of the SROC curve, which ranges from 0.5 (no diagnostic value) to 1.0 (perfect diagnostic value), provides a more comprehensive measure of a biomarker's discriminative ability. The AUC of 0.92 observed for IL-27 in our study confirms its high overall diagnostic value for neonatal sepsis. Given the high morbidity and mortality associated with neonatal sepsis, biomarkers with high sensitivity and NPV are particularly valuable for reducing missed diagnoses during initial triage. While CRP is widely used clinically for early sepsis detection (23–25) our meta-analysis showed that IL-27 outperformed CRP in both sensitivity (82% vs. 73%) and specificity (85% vs. 76%), with a higher AUC (0.92 vs. 0.84). The 9% higher pooled sensitivity of IL-27 translates to improved rule-out capability, which could minimize delayed intervention in critically ill neonates—especially in cases where traditional markers remain equivocal in the early stages of infection.

A major barrier to the clinical translation of IL-27 is the lack of a standardized diagnostic cutoff value. An additional, equally critical obstacle is the absence of a rapid turnaround test for IL-27. Unlike CRP, which is widely available as a point-of-care or real-time assay in most clinical settings, IL-27 currently requires ELISA-based methods that are time-consuming and not suitable for emergency decision-making. Until a rapid assay becomes available, the clinical benefit of IL-27—despite its superior diagnostic accuracy—remains speculative, as timely initiation of therapy in neonatal sepsis cannot await delayed test results. Even with the same assay, reported IL-27 cutoffs for neonatal sepsis vary widely (283.8–1,000 pg/mL) across studies, as observed in our analysis. This variability may stem from differences in sepsis severity, study design, clinical settings (e.g., resource-rich vs. resource-limited environments), and sample types (26). Standardizing IL-27 measurement protocols and establishing context-specific cutoff values—accounting for factors such as gestational age, postnatal age, and sepsis onset type (EOS vs. LOS)—are essential for its widespread clinical application. Furthermore, pretest probabilities vary between EOS and LOS, and this discrepancy may contribute to the observed differences in IL-27′s diagnostic performance between these two subgroups (Table 2). Future studies should prioritize addressing these confounding factors to refine the clinical utility of IL-27.

This meta-analysis has several limitations that should be considered when interpreting the results. First, substantial heterogeneity was observed across key diagnostic metrics (e.g., sensitivity *I*^2^ = 92.0%, specificity *I*^2^ = 83.6%). While subgroup analyses based on sepsis onset type and geographic region identified potential sources of heterogeneity, some residual heterogeneity remains unexplained. Second, only five studies were included in the final analysis, reflecting the limited availability of high-quality data on IL-27's diagnostic performance in neonatal sepsis. Third, statistically significant publication bias was detected (*P* < 0.05), which may have led to overrepresentation of studies with positive results. Fourth, all included studies measured IL-27 using ELISA or similar batch assays with turnaround times incompatible with real-time clinical decision-making. Therefore, while our meta-analysis confirms the diagnostic accuracy of IL-27, its actual clinical utility in guiding immediate management of suspected neonatal sepsis remains to be demonstrated once rapid testing platforms become available. Fifth, our meta-analysis only included studies that defined neonatal sepsis on the basis of positive blood culture. However, a substantial proportion of neonatal sepsis cases are culture-negative or viral in origin (27, 28). Therefore, our findings regarding the diagnostic accuracy of IL-27 apply specifically to culture-confirmed bacterial sepsis and may not be generalizable to culture-negative or viral sepsis. Future studies should evaluate the performance of IL-27 in these understudied populations (8). These factors collectively introduce a risk of bias and may limit the generalizability of our findings.

In conclusion, the current evidence indicates that IL-27 is a highly valuable biomarker for the diagnosis of culture-confirmed bacterial neonatal sepsis, with superior diagnostic accuracy compared to CRP. However, these findings should be interpreted with caution due to the small number of included studies and limited overall sample size, which may reduce the reliability and generalizability of the results. To fully establish the clinical utility of IL-27 and validate our findings, large-sample, multicenter, prospective diagnostic accuracy studies are urgently warranted. Future research should also focus on establishing standardized IL-27 measurement protocols, defining optimal age- and context-specific cutoff values, and validating its diagnostic performance in diverse neonatal populations (e.g., preterm vs. term infants, different geographic regions).

Importantly, the development and clinical validation of a rapid turnaround IL-27 assay is a prerequisite for translating its superior diagnostic accuracy into tangible bedside benefits; until then, its role in real-time sepsis management remains theoretical. Large-scale investigations addressing these gaps will not only help determine the optimal sample size required for robust clinical application but also strengthen the reliability of IL-27 as a diagnostic tool, ultimately contributing to improved clinical outcomes for neonates at risk of bacterial sepsis.

## Data Availability

The original contributions presented in the study are included in the article/Supplementary Material, further inquiries can be directed to the corresponding author.
