# Supplementary material for: Diagnostic performance of interleukin-27 and C-reactive protein in neonatal sepsis: an updated systematic review and meta-analysis
Source: Front Pediatr. 2026 May 13;14:1781391. doi: 10.3389/fped.2026.1781391 (PMC13212332; doi:10.3389/fped.2026.1781391)
Supplement: Supplementary file 1 [file Supplementaryfile1.docx]

Supplement 1. The search strategy in pubmed.

#1 ((("Interleukin-27"[Mesh]) OR Interleukin-27 [Title/Abstract]) OR IL-27 [Title/Abstract])

#2 ((((((((((((((((("Neonatal Sepsis"[Mesh]) OR Neonatal Sepsis[Title/Abstract]) OR Sepsis, Neonatal[Title/Abstract]) OR Sepsis, Neonatal[Title/Abstract]) OR Neonatal Late-Onset Sepsis[Title/Abstract]) OR Late-Onset Sepsis, Neonatal[Title/Abstract]) OR Late-Onset Sepsis, Neonatal[Title/Abstract]) OR Neonatal Late Onset Sepsis[Title/Abstract]) OR Neonatal Late-Onset Sepsis[Title/Abstract]) OR Sepsis, Neonatal Late-Onset[Title/Abstract]) OR Sepsis, Neonatal Late-Onset[Title/Abstract]) OR Neonatal Early-Onset Sepsis[Title/Abstract]) OR Early-Onset Sepsis, Neonatal[Title/Abstract]) OR Early-Onset Sepsis, Neonatal[Title/Abstract]) OR Neonatal Early Onset Sepsis[Title/Abstract]) OR Neonatal Early-Onset Sepsis[Title/Abstract]) OR Sepsis, Neonatal Early-Onset[Title/Abstract]) OR Sepsis, Neonatal Early-Onset[Title/Abstract]

#3 #1 AND #2


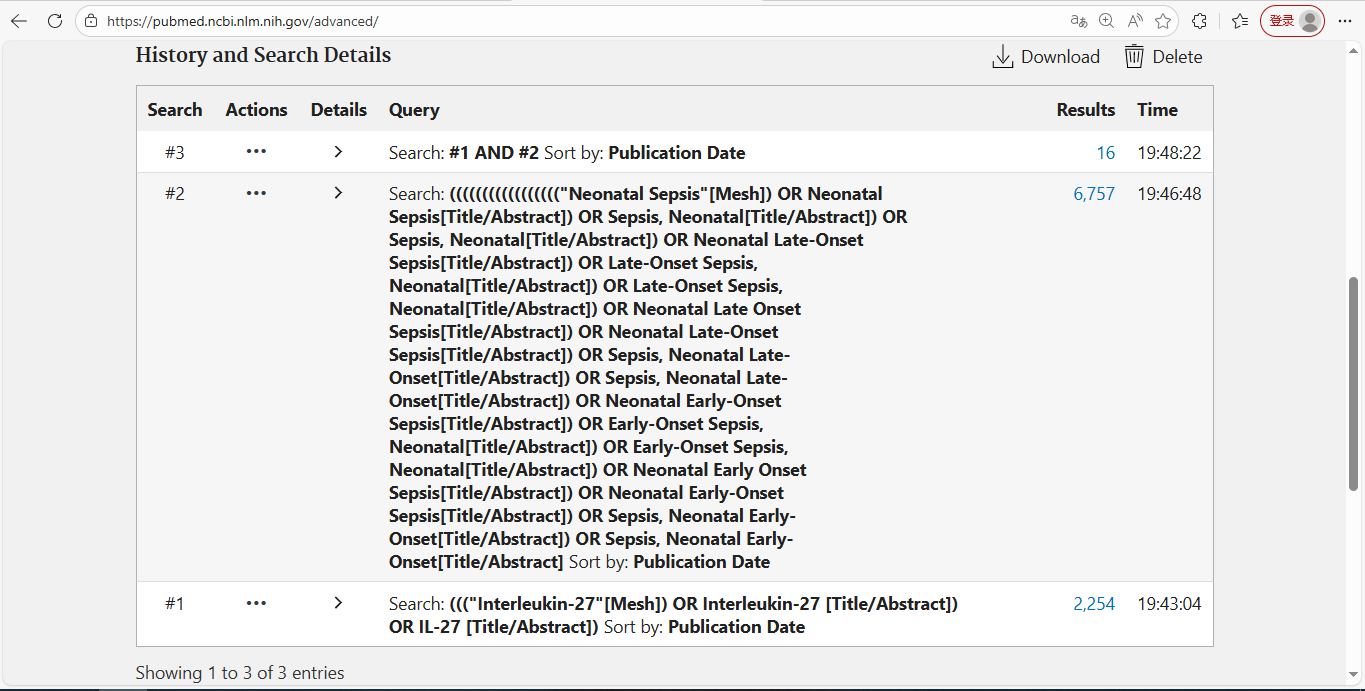


Supplement 2. The search strategy in the Cochrane library.

#1 ((Interleukin-27) OR IL-27)

#2 (Neonatal Sepsis) OR (Sepsis, Neonatal) OR (Sepsis, Neonate) OR (Neonatal Late-Onset Sepsis) OR (Late-Onset Sepsis, Neonatal) OR (Late-Onset Sepsis, Neonate) OR (Neonatal Early-Onset Sepsis) OR (Early-Onset Sepsis, Neonatal) OR (Early-Onset Sepsis, Neonate) OR (Neonatal Early Onset Sepsis) OR (Sepsis, Neonatal Early-Onset)

#3 #1 AND #2


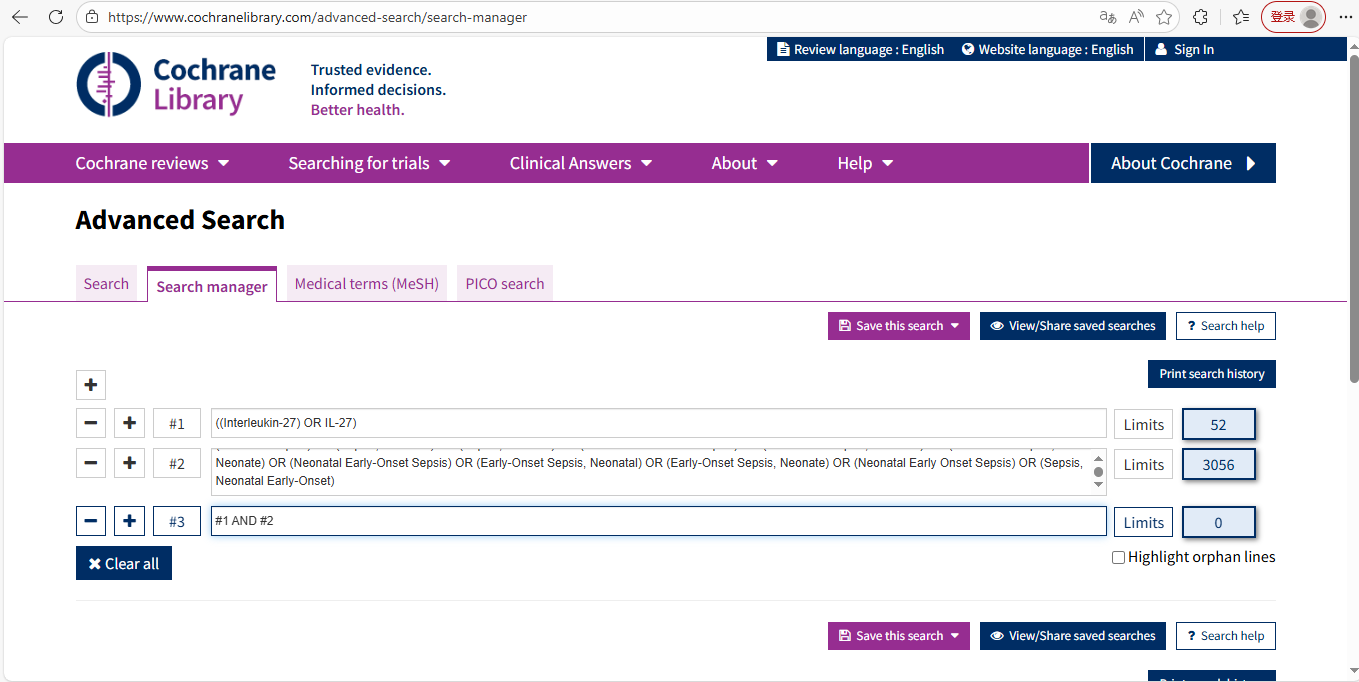


Supplement 3. The search strategy in Embase.

((Interleukin-27) OR IL-27) and ((Neonatal Sepsis) OR (Sepsis, Neonatal) OR (Sepsis, Neonate) OR (Neonatal Late-Onset Sepsis) OR (Late-Onset Sepsis, Neonatal) OR (Late-Onset Sepsis, Neonate) OR (Neonatal Early-Onset Sepsis) OR (Early-Onset Sepsis, Neonatal) OR (Early-Onset Sepsis, Neonate) OR (Neonatal Early Onset Sepsis) OR (Sepsis, Neonatal Early-Onset))/br


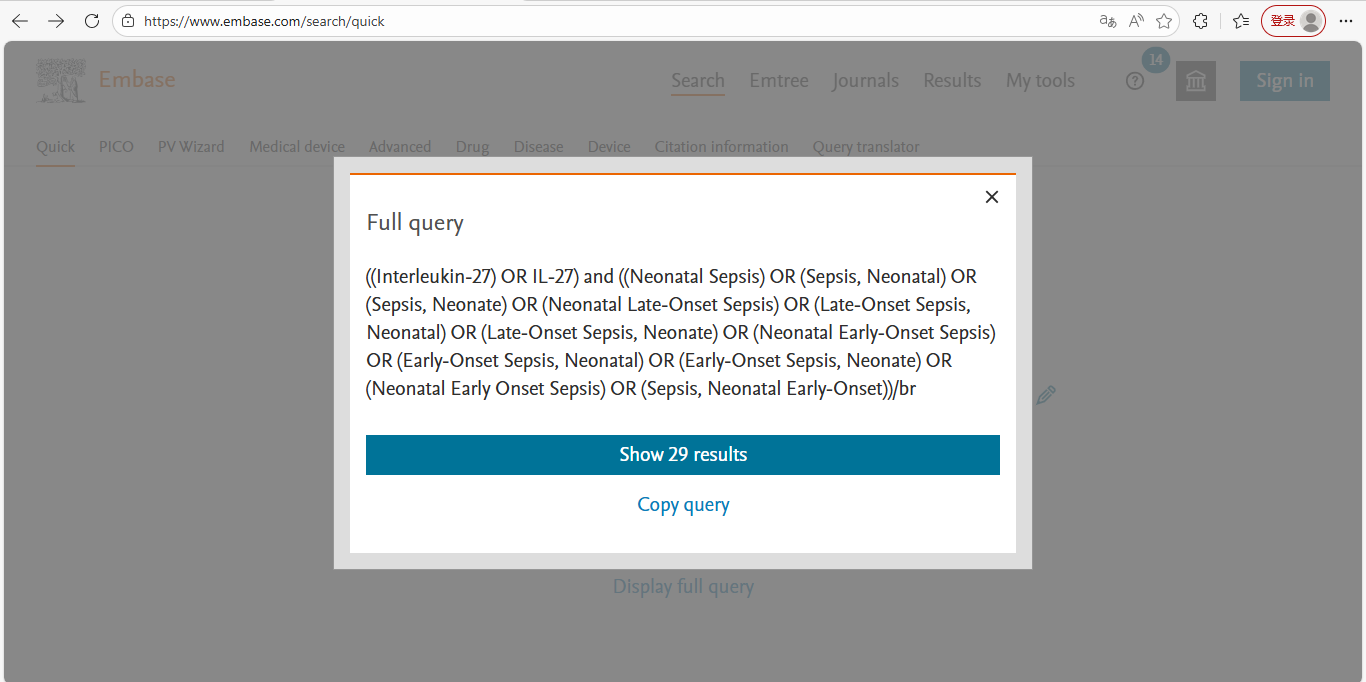


Supplement 4.

**Summary Sensitivity**

Study | Sen [95% Conf. Iterval.] TP/(TP+FN) TN/(TN+FP)

--------------------------------------------------------------------------------------

Rautela 2024 | 0.681 0.529 - 0.809 32/47 24/33

Tosson 2024 | 1.000 0.920 - 1.000 44/44 45/46

Fahmy 2020 | 0.863 0.737 - 0.943 44/51 30/33

Abo 2018 | 1.000 0.918 - 1.000 43/43 45/47

He 2017 | 0.667 0.546 - 0.773 48/72 59/79

--------------------------------------------------------------------------------------

**Pooled Sen | 0.821 0.769 - 0.866**

--------------------------------------------------------------------------------------

Heterogeneity chi-squared = 50.19 (d.f.= 4) p = 0.000

Inconsistency (I-square) = 92.0 %

No. studies = 5.

Filter OFF

Add 1/2 to all cells of the studies with zero

**Summary Specificity**

Study | Spe [95% Conf. Iterval.] TP/(TP+FN) TN/(TN+FP)

--------------------------------------------------------------------------------------

Rautela 2024 | 0.727 0.545 - 0.867 32/47 24/33

Tosson 2024 | 0.978 0.885 - 0.999 44/44 45/46

Fahmy 2020 | 0.909 0.757 - 0.981 44/51 30/33

Abo 2018 | 0.957 0.855 - 0.995 43/43 45/47

He 2017 | 0.747 0.636 - 0.838 48/72 59/79

--------------------------------------------------------------------------------------

**Pooled Spe | 0.853 0.801 - 0.895**

--------------------------------------------------------------------------------------

Heterogeneity chi-squared = 24.42 (d.f.= 4) p = 0.000

Inconsistency (I-square) = 83.6 %

No. studies = 5.

Filter OFF

Add 1/2 to all cells of the studies with zero

**Summary Positive Likelihood Ratio (Random effects model)**

Study | LR+ [95% Conf. Iterval.] % Weight

--------------------------------------------------------------------------------------

Rautela 2024 | 2.496 1.383 - 4.506 23.15

Tosson 2024 | 30.985 6.415 - 149.66 15.19

Fahmy 2020 | 9.490 3.208 - 28.071 19.24

Abo 2018 | 18.982 5.676 - 63.483 18.19

He 2017 | 2.633 1.743 - 3.978 24.22

--------------------------------------------------------------------------------------

**(REM) pooled LR+ | 6.934 2.627 - 18.301**

--------------------------------------------------------------------------------------

Heterogeneity chi-squared = 28.38 (d.f.= 4) p = 0.000

Inconsistency (I-square) = 85.9 %

Estimate of between-study variance (Tau-squared) = 0.9681

No. studies = 5.

Filter OFF

Add 1/2 to all cells of the studies with zero

**Summary Negative Likelihood Ratio (Random effects model)**

Study | LR- [95% Conf. Iterval.] % Weight

--------------------------------------------------------------------------------------

Rautela 2024 | 0.439 0.275 - 0.700 27.43

Tosson 2024 | 0.011 0.001 - 0.181 9.40

Fahmy 2020 | 0.151 0.075 - 0.303 25.65

Abo 2018 | 0.012 0.001 - 0.189 9.40

He 2017 | 0.446 0.314 - 0.634 28.13

--------------------------------------------------------------------------------------

**(REM) pooled LR- | 0.170 0.061 - 0.474**

--------------------------------------------------------------------------------------

Heterogeneity chi-squared = 36.50 (d.f.= 4) p = 0.000

Inconsistency (I-square) = 89.0 %

Estimate of between-study variance (Tau-squared) = 0.9443

No. studies = 5.

Filter OFF

Add 1/2 to all cells of the studies with zero

**Summary Diagnostic Odds Ratio (Random effects model)**

Study | DOR [95% Conf. Iterval.] % Weight

--------------------------------------------------------------------------------------

Rautela 2024 | 5.689 2.132 - 15.178 23.85

Tosson 2024 | 2699.7 107.09 - 68054.6 14.34

Fahmy 2020 | 62.857 15.044 - 262.64 22.17

Abo 2018 | 1583.4 73.895 - 33928.6 14.98

He 2017 | 5.900 2.915 - 11.943 24.66

--------------------------------------------------------------------------------------

**(REM) pooled DOR | 54.973 8.706 - 347.13**

--------------------------------------------------------------------------------------

Heterogeneity chi-squared = 34.56 (d.f.= 4) p = 0.000

Inconsistency (I-square) = 88.4 %

Estimate of between-study variance (Tau-squared) = 3.4555

No. studies = 5.

Filter OFF

Add 1/2 to all cells of the studies with zero
